# Cerebral Fat Macroembolism: Diagnosic Tools with Spectral Imaging

**DOI:** 10.5334/jbsr.3811

**Published:** 2024-12-17

**Authors:** Mathilde Haegeman, Douglas Lacomblez, Antoine Loubet

**Affiliations:** 1 Radiology Department, UCL Bruxelles, Cliniques Universitaires Saint-Luc, Belgium Avenue Hippocrate 10, 1200 Bruxelles, Belgium

**Keywords:** Spectral CT, Z-effective map, stroke, cerebral fat embolism

## Abstract

*Teaching point:* Spectral tomography offers valuable complementary diagnostic tools in the setting of cerebral fat macroembolism, a rare condition often presenting with nonspecific clinical symptoms.

## Case History

An 84‑year‑old man was referred to the surgical unit for total shoulder replacement after a fracture of the left humerus. During surgery, the electroencephalogram showed bilateral cerebral hypoperfusion and severe arterial hypotension. Post‑operatively, the patient presented with altered level of consciousness, suggesting a stroke.

A brain computed tomography (CT) scan was performed on a spectral CT system with dual‑layer detectors for low‑ and high‑energy photon capture (IQON CT, Philips Healthcare, Cleveland, OH, USA), with intravenous iodinated contrast (Xenetix 350, Guerbet).

**Figure 1:**
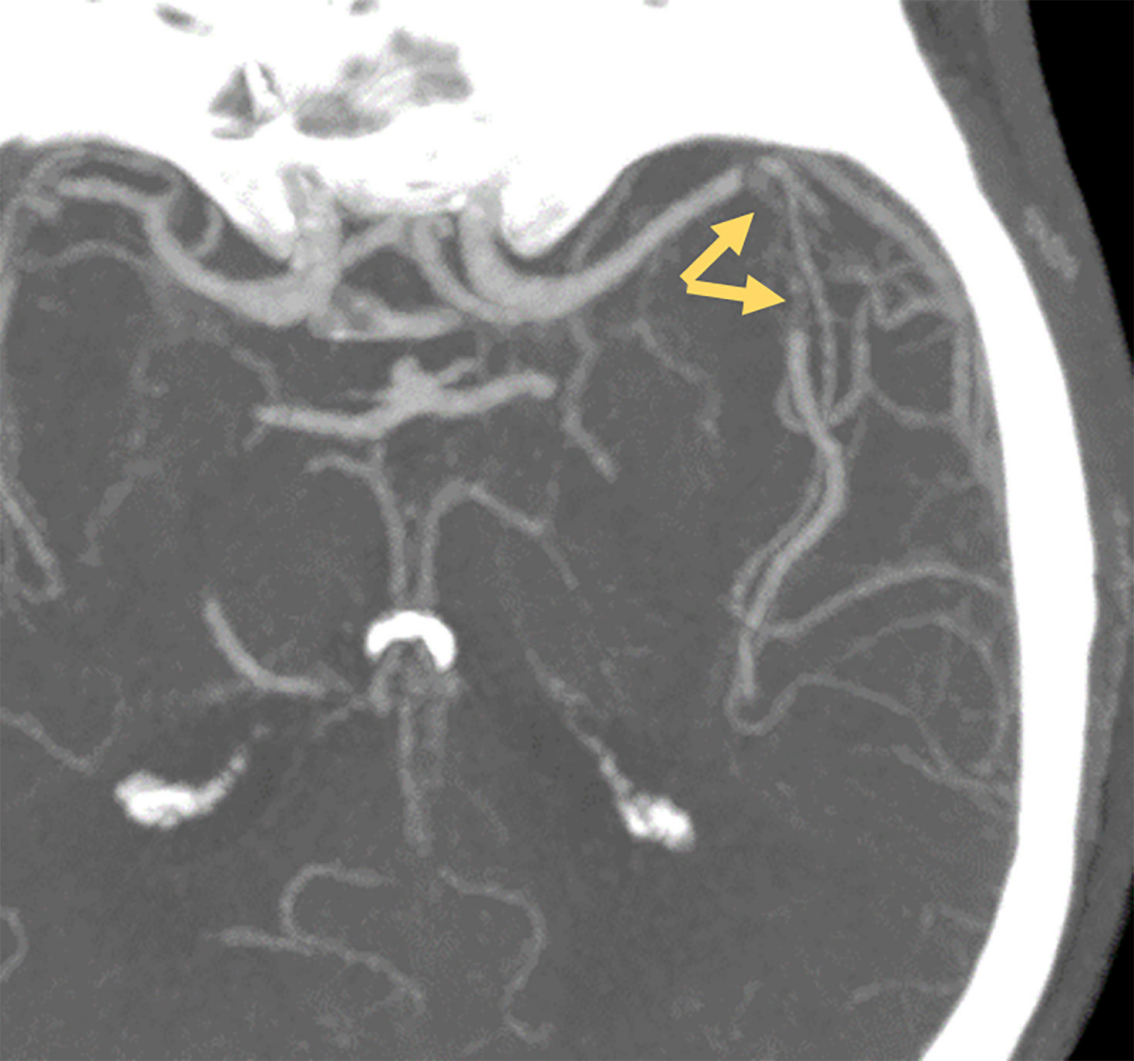
Axial CT scan showing a sub‑occlusive embolus of the M2 portion of the left middle cerebral artery (arrows) with downstream recanalization.

The CT scan revealed no parenchymal abnormalities. However a supracentimetric subocclusive macro‑embolus was observed in the M2 segment of the left middle cerebral artery (MCA) with downstream recanalisation (Figure 1) and an occlusion of the distal part of a right M3 branch. Cerebral perfusion showed multifocal hypoperfusion without a core.

**Figure 2:**
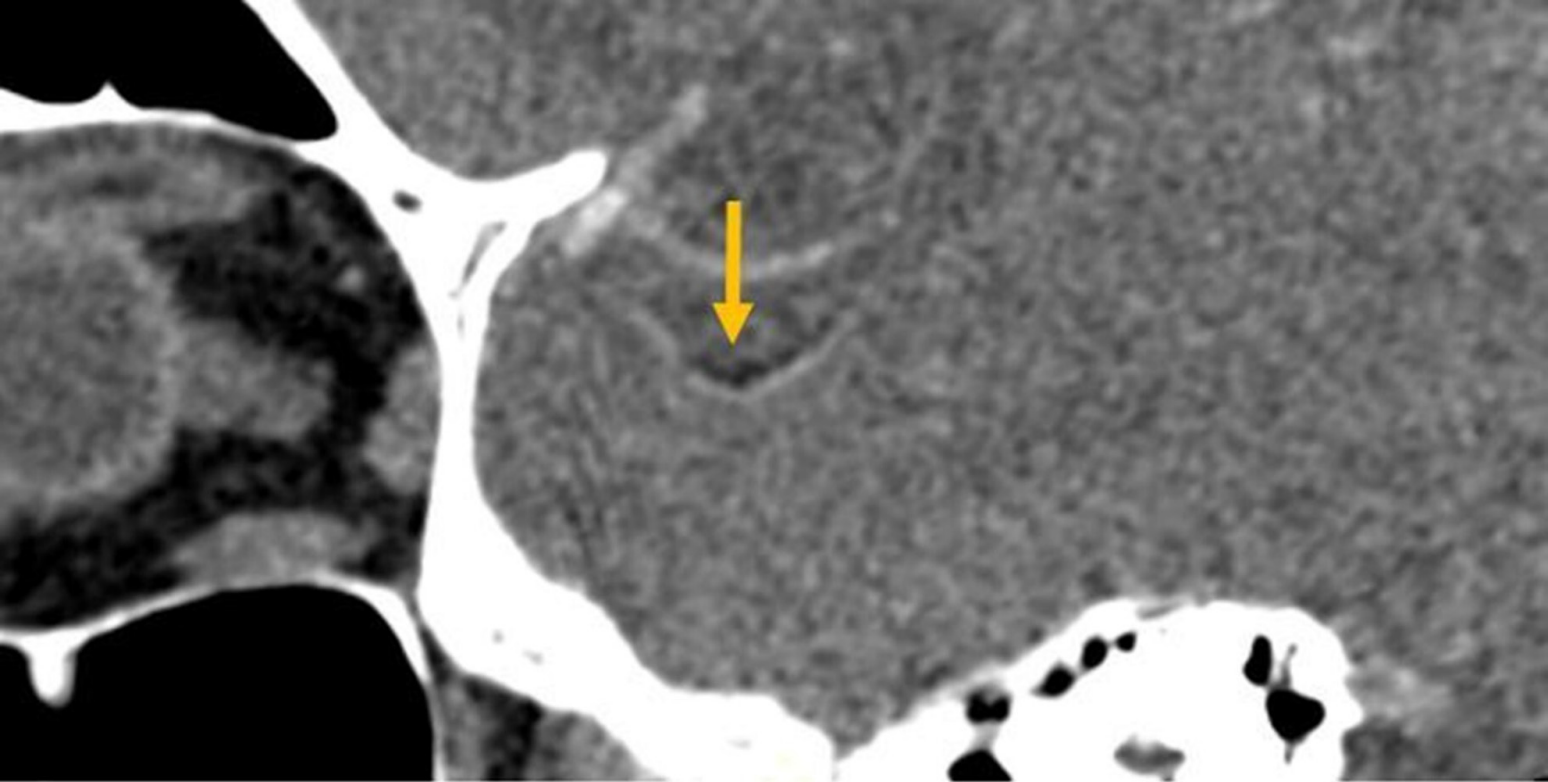
An MPR CT view of the CTA showing the sub‑occlusive embolus of the M2 portion of the left middle cerebral artery with very low density (arrow).

**Figure 3:**
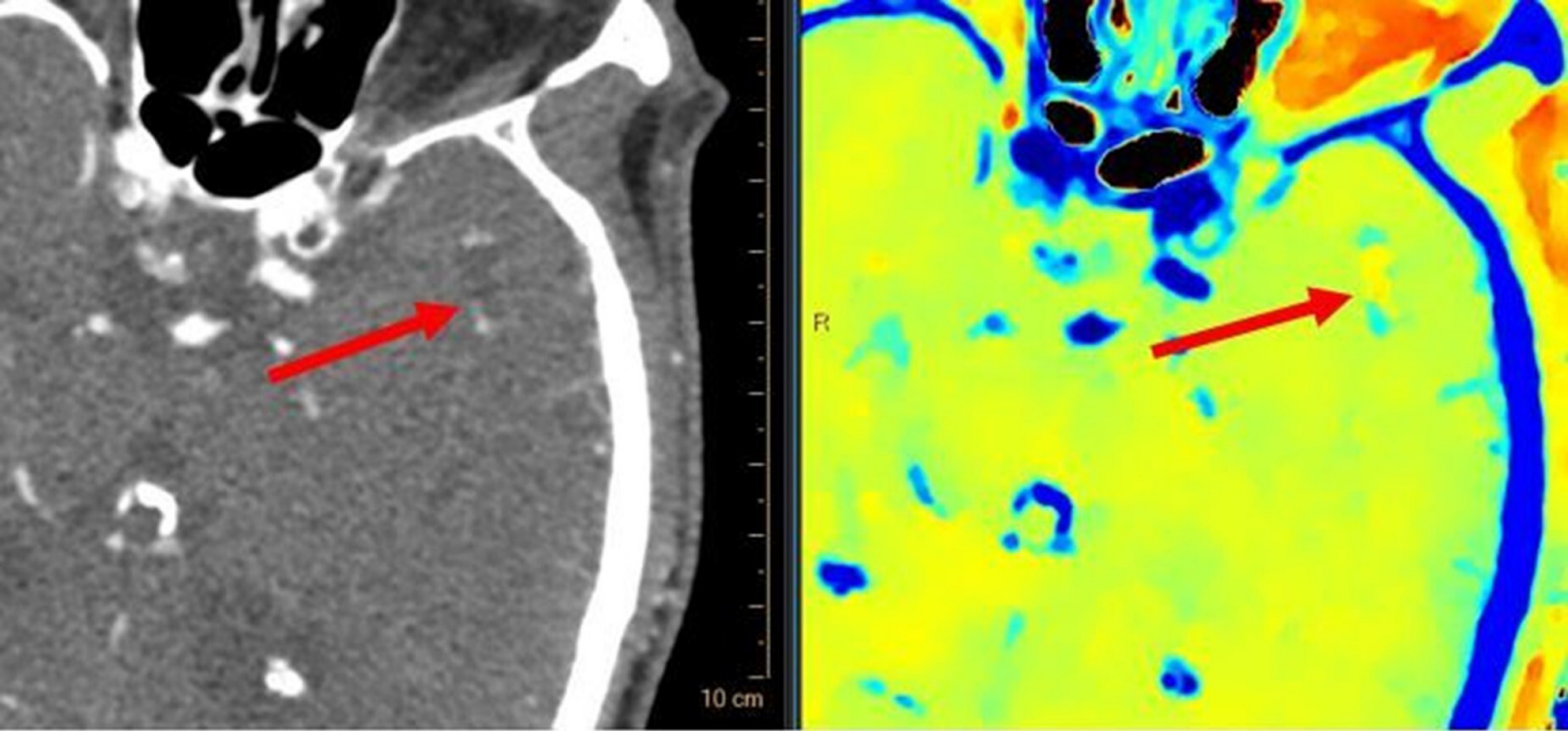
On the right: Spectral Z‑effective map showing fatty density (orange/red) of the embolus of the M2 portion of the left middle cerebral artery (arrows). On the left: corresponding CTA.

On the late‑phase Computed tomography angiography acquisition (obtained 24.1 s after arterial phase), the embolus in the M2 segment of the left MCA showed a markedly low density (Figure 2), also observed on the unenhanced CT scan. To confirm the suspicion of a fat embolus, the *Z*‑effective spectral map was used (Figure 3), which indicated fat density (shown in orange/red on the proprietary colour scale).

Mechanical thrombectomy (MT) of the M2 segment of the left MCA was successfully performed, and histological analysis of the embolus confirmed its fatty composition.

## Discussion

*Z*‑effective mapping technology, based on spectral CT acquisition, is used to characterise voxels based on their effective atomic number (*Z*) and electron density, giving the mean atomic values of the tissue. In the available system, this scale is converted into a range of colours representing mean atomic tissue numbers from 6 (or lower) to 11 (or higher).

Fatty embolism is primarily diagnosed clinically, often using Gurd’s criteria, including respiratory distress, neurological involvement and petechial rash. However, these symptoms are not always present. Neurological manifestations are common but are largely variable and non‑specific [1]. In general, fat emboli are small, multifocal and occur in distal branches and, therefore, are not easily visible on CT. Magnetic resonance imaging (MRI) is the preferred imaging for early and accurate diagnosis.

In cases of macroscopic fat embolism, such as in the case presented, where there is proximal obstruction of an intracranial vessel, *Z*‑effective mapping offers complementary information regarding the fat content of the embolus. Identifying the fat embolus allows for timely and appropriate intervention, such as mechanical thrombectomy. Intravenous thrombolysis would be ineffective due to the non‑thrombotic composition of the embolism.

Spectral imaging shows promising potential for applications in neuroimaging, though further studies are needed to assess its full role in stroke management, particularly its potential applications in characterising the content of intracerebral emboli.
